# ApoA2 isoforms as blood-based biomarkers reflecting pancreatic exocrine dysfunction for risk stratification and perioperative management of pancreatic cancer

**DOI:** 10.3389/fonc.2026.1902440

**Published:** 2026-09-17

**Authors:** Akira Matsushita, Takashi Ono, Takahiro Murokawa, Junji Ueda, Tetsuya Shimizu, Yoichi Kawano, Akihisa Matsuda, Yuta Hasegawa, Norio Itokawa, Keiko Takeuchi, Ayumi Kashiro, Kazufumi Honda

**Affiliations:** 1 Department of Gastroenterological Surgery, Nippon Medical School, Tokyo, Japan; 2 Department of Gastroenterology, Nippon Medical School, Tokyo, Japan; 3 Department of Molecular Prevention, Graduate School of Medicine, Nippon Medical School, Tokyo, Japan; 4 Division of Molecular Prevention, Institute for Advanced Medical Science, Nippon Medical School, Tokyo, Japan

**Keywords:** apolipoprotein A2 isoforms, intraductal papillary mucinous neoplasms, new-onset diabetes, pancreatic adenocarcinoma, pancreatic exocrine insufficiency, pancreatic fistula, steatotic liver disease, type 3C diabetes mellitus

## Abstract

Apolipoprotein A2 isoforms (apoA2‑i) are clinically validated blood‑based biomarkers for the auxiliary diagnosis of pancreatic cancer. Emerging evidence also suggests that apoA2‑i may contribute to the detection of early‑stage pancreatic cancer, particularly in cohorts where CA19‑9 shows limited sensitivity, although these applications remain investigational and require prospective validation. Beyond diagnostic applications, apoA2‑i reflect pancreatic exocrine physiology, linking biomarker biology with perioperative outcomes such as postoperative pancreatic fistula and steatotic liver disease. Because type 3c diabetes mellitus arises from exocrine dysfunction, emerging evidence suggests that exocrine‑based markers, including apoA2‑i, may contribute to future approaches for stratifying new‑onset diabetes, although current evidence remains preliminary. By integrating validated diagnostic roles with developing translational and physiological applications, this review outlines the evolving relevance of apoA2‑i within pancreatic oncology while emphasizing the need for prospective, multi-center validation and real-world data integration.

## 1 Introduction

Pancreatic ductal adenocarcinoma (PDAC) remains one of the most lethal malignancies, with a 5‑year survival rate of only 12.5% despite advances in systemic therapy and surgical techniques (1). Most patients are diagnosed at an advanced stage because early disease is clinically silent and the pancreas is anatomically concealed in the retroperitoneum (2). Curative resection offers the only chance for long‑term survival, yet fewer than 20% of patients are eligible at diagnosis, underscoring the need for strategies that enable detection at surgically resectable stages.

PDAC develops through a multistep evolutionary process initiated by activating *KRAS* mutations, followed by recurrent alterations in *TP53*, *CDKN2A*, and *SMAD4*, which define distinct molecular subtypes and shape tumor behavior (3, 4). The tumor microenvironment is characterized by dense desmoplastic stroma and heterogeneous cancer‑associated fibroblast populations that promote immune evasion and metabolic reprogramming (5). PDAC cells adapt to nutrient‑poor conditions through *KRAS*‑driven glutamine metabolism and macropinocytosis (6, 7), while suppressive myeloid populations dominate the immune landscape (8). Precursor lesions such as PanIN and intraductal papillary mucinous neoplasm (IPMN) evolve through stepwise genetic progression over many years, providing a biological window for early detection (9).

Despite this prolonged preclinical phase, current diagnostic pathways rely heavily on imaging modalities such as MRCP, EUS, and ERCP, which are typically performed only after symptom onset. The limitations of carbohydrate antigen 19‑9 (CA19‑9), including low sensitivity for early‑stage disease, false-negative results in Lewis antigen–negative individuals, and elevated levels in benign conditions, further restrict its ability to detect PDAC at a curable stage (10). These challenges have accelerated interest in cancer interception strategies (11) and multi‑analyte blood tests capable of detecting surgically resectable cancers (12).

Among emerging candidates, apolipoprotein A2 isoforms (apoA2‑i) have shown consistent promise across multiple independent studies. A large multicenter clinical performance study demonstrated that apoA2‑ATQ/AT provides strong discriminatory ability for (13), the detection of pancreatic cancer, including early‑stage disease in selected cohorts, although these findings remain investigational and require further prospective validation (13), with subsequent studies confirming its complementary value alongside CA19‑9 (14) and validating its reproducibility in real‑world settings (15).

Recent translational studies have expanded the relevance of apoA2‑i beyond initial diagnostic assessment. ApoA2‑i have shown preliminary ability to stratify malignant potential in IPMNs, and plasma apoA2‑ATQ/AT provides a tumor‑related proteolytic signature that complements imaging‑based surveillance (16). In addition, plasma apoA2‑AT/AT levels reflect pancreatic exocrine function and predict postoperative outcomes, including new‑onset steatotic liver disease following pancreatectomy (17) and postoperative pancreatic fistula after distal pancreatectomy (18). Together, these findings indicate that apoA2‑i may reflect both tumor‑associated microenvironmental changes and exocrine physiological alterations across the spectrum of pancreatic disease.

This review synthesizes current biological, diagnostic, and translational evidence on apoA2‑i and proposes a balanced overview for their application across pancreatic oncology, from diagnostic evaluation, including early‑stage disease, to risk stratification and perioperative management.

## 2 Biological basis of pancreatic ductal adenocarcinoma

Pancreatic ductal adenocarcinoma (PDAC) develops through a multistep evolutionary process driven by early oncogenic activation, progressive genomic instability, and profound remodeling of the pancreatic microenvironment. More than 9 of PDACs harbor activating *KRAS* mutations, which initiate aberrant MAPK and PI3K signaling, induce acinar‑to‑ductal metaplasia (ADM), and reprogram cellular metabolism (2). Comprehensive genomic analyses have further revealed distinct molecular subtypes, classical and basal‑like, each associated with unique transcriptional programs and clinical behavior (3). Integrated genomic profiling by The Cancer Genome Atlas has confirmed recurrent alterations in *TP53*, *CDKN2A*, and *SMAD4*, which collectively disrupt cell‑cycle regulation, DNA damage responses, and TGF‑β signaling (4). These sequential genetic events underpin the progression from low‑grade precursor lesions to invasive carcinoma.

The PDAC tumor microenvironment is dominated by dense desmoplastic stroma composed of heterogeneous cancer‑associated fibroblast (CAF) populations. Landmark single‑cell and functional studies have identified two major CAF subsets, inflammatory CAFs and myofibroblastic CAFs, each exerting distinct effects on tumor progression, cytokine signaling, and extracellular matrix remodeling (5). This fibroblast heterogeneity contributes to the profound stromal expansion and therapeutic resistance characteristic of PDAC.

Metabolic reprogramming represents another hallmark of PDAC biology. Oncogenic *KRAS* drives extensive metabolic rewiring, including enhanced glycolysis and altered glutamine utilization (6). In addition, PDAC cells rely on macropinocytosis to scavenge extracellular proteins as alternative nutrient sources, enabling anabolic growth under nutrient‑poor conditions—a phenomenon demonstrated in a landmark study showing that PDAC tumors are profoundly nutrient deprived and depend on extracellular protein uptake for survival (7).

PDAC also exhibits a profoundly immunosuppressive microenvironment. Myeloid‑derived suppressor cells and tumor‑associated macrophages dominate the immune landscape, restricting effector T‑cell infiltration. A pivotal study demonstrated that myeloid cells are required for PD‑1/PD‑L1 checkpoint activation and for establishing the immunosuppressive tumor microenvironment characteristic of PDAC (8).

Importantly, phylogenetic and genomic timing analyses have demonstrated that PDAC evolves over a prolonged preclinical period. Low‑grade precursor lesions accumulate sequential driver alterations and remain clinically silent for many years before acquiring metastatic competence (9). This extended evolutionary window provides a biological rationale for early detection and highlights the need for biomarkers capable of capturing systemic alterations, including metabolic, immunologic, and exocrine changes, associated with early tumorigenesis (**Figure 1**).

**Figure 1:**
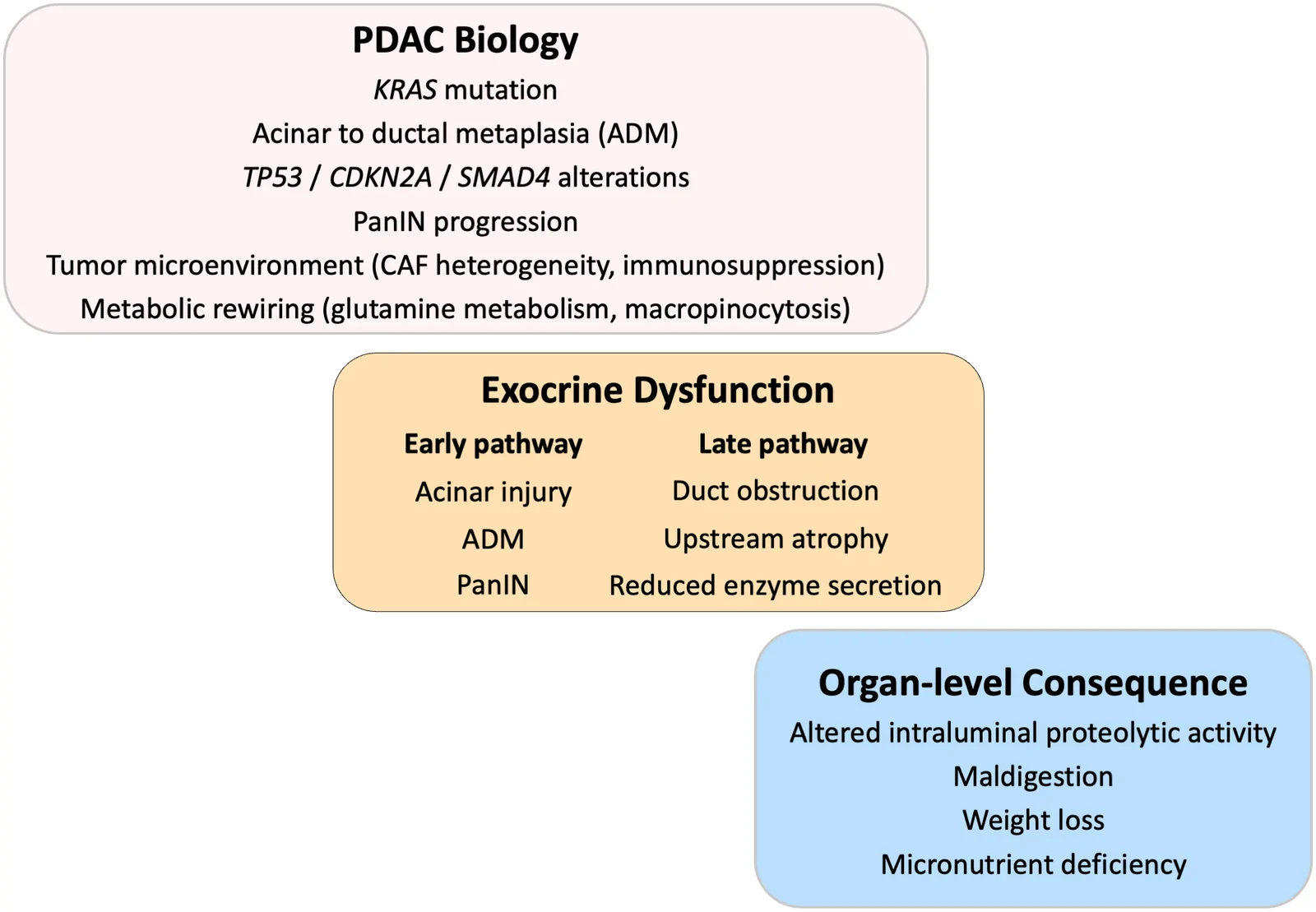
Biological pathways linking pancreatic ductal adenocarcinoma (PDAC) development with pancreatic exocrine dysfunction. PDAC arises through early oncogenic events—including *KRA*S‑driven acinar‑to‑ductal metaplasia (ADM), sequential alterations in *TP53, CDKN2A*, and S*MAD4*, and progression through PanIN lesions—accompanied by stromal remodeling, metabolic rewiring, and immune suppression. These tumor‑intrinsic and microenvironmental changes generate two complementary pathways of exocrine dysfunction. The early pathway reflects acinar injury and ADM‑associated loss of exocrine identity, whereas the late pathway results from tumor‑induced ductal obstruction leading to upstream atrophy and reduced digestive enzyme secretion. Together, these processes alter intraluminal proteolytic activity and contribute to systemic consequences such as maldigestion, weight loss, and micronutrient deficiency.

## 3 Exocrine dysfunction and ApoA2 isoform processing in pancreatic tumorigenesis

Progressive impairment of pancreatic exocrine function arises through two biologically complementary mechanisms during PDAC evolution. First, acinar cell injury and inflammation can trigger ADM and PanIN formation, linking disruption of exocrine cell identity to early tumorigenesis (19). Experimental models demonstrate that *KRAS* activation in acinar cells, particularly in the context of inflammatory stress, drives reprogramming toward ductal neoplasia, establishing exocrine dysfunction as an early organ‑level phenotype associated with tumor initiation (20). Second, once a tumor is established, progressive ductal obstruction leads to upstream acinar atrophy, loss of zymogen granules, and reduced secretion of digestive enzymes. Recent European guidelines emphasize that pancreatic ductal obstruction caused by pancreatic cancer is a major mechanism leading to exocrine insufficiency, reflecting both loss of functional exocrine tissue and impaired intraluminal enzyme activity (21). These tumor‑induced alterations amplify inflammatory and metabolic remodeling within the organ environment and contribute to systemic manifestations such as maldigestion, weight loss, and micronutrient deficiencies.

Together, these dual pathways, exocrine dysfunction as both a driver and a consequence of tumorigenesis, generate systemic biochemical signatures that may precede radiologic detectability. Genomic timing studies indicate that precursor lesions accumulate driver alterations over several years before acquiring metastatic competence, providing a prolonged evolutionary window during which organ‑level dysfunction and its circulating correlates may already be present. Together, these biological characteristics underscore the difficulty of early PDAC detection and highlight the need for minimally invasive biomarkers capable of capturing disease‑associated systemic and exocrine changes (**Figure 1**).

These dual pathways of exocrine dysfunction, arising both as an early consequence of acinar injury and as a downstream effect of tumor‑induced ductal obstruction, result in measurable alterations in digestive enzyme secretion and intraluminal proteolytic activity. Proteomic studies have shown that apolipoprotein A2 (apoA2) circulates as multiple dimeric isoforms that differ at their C‑terminal residues (ATQ/ATQ, ATQ/AT, AT/AT, AT/A, and A/A), and that these isoform patterns are altered in pancreatic diseases, including pancreatic cancer and chronic pancreatitis (22). Thus, the biological processes that shape PDAC initiation, progression, and exocrine impairment naturally give rise to circulating apoA2‑i signatures, providing a mechanistic rationale for their use as blood‑based biomarkers across early detection and risk stratification.

Unique alterations in the C‑terminal amino acids of circulating apoA2 homodimers give rise to two characteristic processing patterns that reflect pancreatic exocrine function. Notably, the intermediate isoform apoA2‑ATQ/AT is not directly measured but is calculated from the concentrations of apoA2‑ATQ and apoA2‑AT, whereas the heavy (ATQ/ATQ) and light (AT/AT) isoforms are directly quantified by ELISA (23). Honda et al (23). demonstrated that pancreatic ductal adenocarcinoma (PDAC) and high‑risk individuals exhibit two patterns: (1) a hyperprocessing pattern, in which the light isoform apoA2‑AT/AT is predominantly observed, indicating enhanced intraluminal proteolytic activity; and (2) a hypoprocessing pattern, characterized by dominance of the heavy isoform apoA2‑ATQ/ATQ, reflecting reduced exocrine enzyme secretion and impaired proteolytic cleavage. Across both patterns, the intermediate isoform apoA2‑ATQ/AT is consistently decreased in PDAC and high‑risk individuals. These two processing patterns form the biological basis for interpreting apoA2‑isoform signatures in pancreatic cancer and exocrine insufficiency (**Figure 2**).

**Figure 2:**
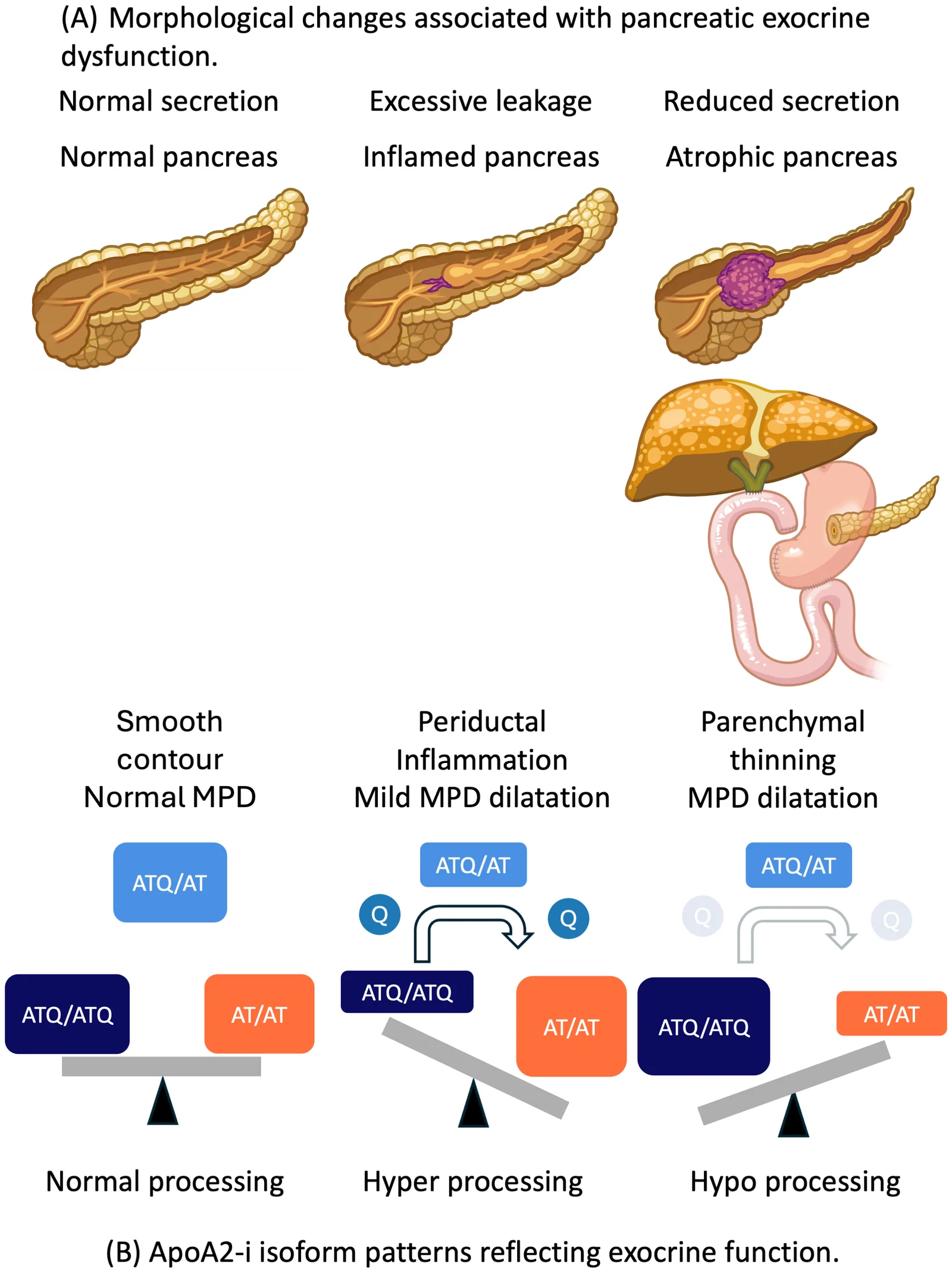
Morphological changes of the pancreas and corresponding apoA2‑i processing patterns. **(A)** *Morphological changes associated with pancreatic exocrine dysfunction.* The normal pancreas shows preserved parenchyma and a thin, uniform main pancreatic duct. Pancreatic duct stenosis with upstream dilatation causes excessive leakage of pancreatic juice. Tumor‑induced distal pancreatic atrophy and postoperative remnant pancreatic atrophy following pancreaticoduodenectomy both lead to reduced exocrine secretion, although the underlying mechanisms differ: tumor obstruction versus surgical resection. **(B)** *ApoA2‑i isoform patterns reflecting exocrine function.* Normal processing produces a balanced distribution of ATQ/ATQ, ATQ/AT, and AT/AT isoforms. Hyperprocessing, reflecting excessive leakage, increases cleavage of ATQ/ATQ and generates more free Q, shown in blue. Hypoprocessing, reflecting reduced secretion, decreases cleavage, resulting in accumulation of ATQ/ATQ and only minimal release of Q, shown in pale blue with 50% transparency. Block height represents the relative abundance of each isoform. *Integrated interpretation.* Morphological abnormalities that increase leakage correspond to hyperprocessing, whereas those that reduce secretion correspond to hypoprocessing. Together, these relationships provide an integrated interpretation in which morphological abnormalities that increase leakage correspond to hyperprocessing, whereas those that reduce secretion correspond to hypoprocessing, allowing apoA2‑i isoform patterns to serve as a functional readout of pancreatic exocrine status.

This mechanistic framework provides a biological basis for interpreting apoA2‑isoform signatures as systemic indicators of exocrine dysfunction and neoplastic risk. These isoform processing patterns also provide the biological foundation for the diverse clinical applications of apoA2‑i. As shown in **Figure 3**, the tumor‑related decrease in ATQ/AT and the exocrine physiology–related shifts in AT/AT map directly onto two distinct categories of clinical utility: diagnostic enrichment for neoplastic disease and functional assessment of pancreatic exocrine reserve.

**Figure 3:**
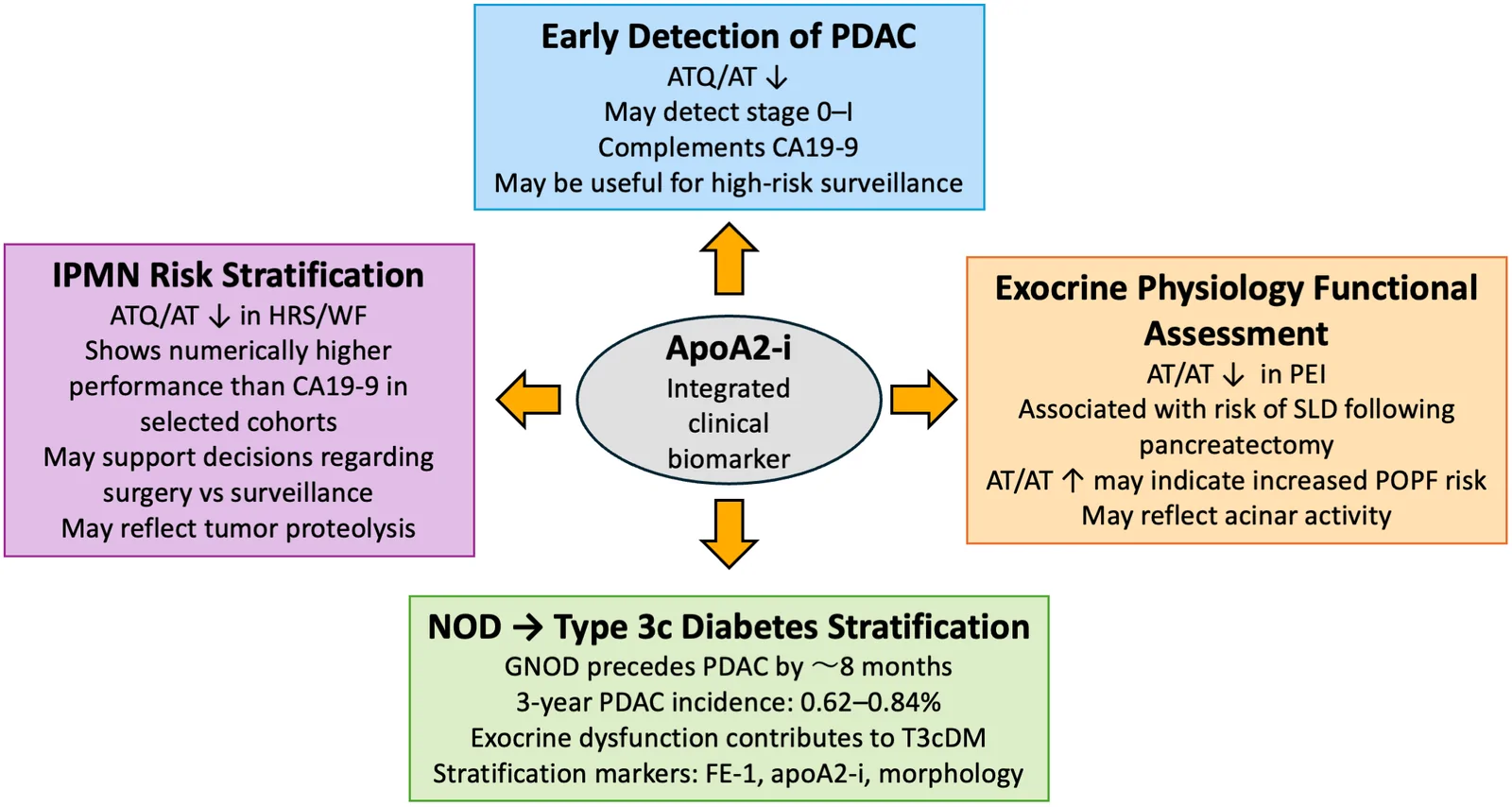
Integrated clinical applications of apolipoprotein A2 isoforms (apoA2‑i) across diagnostic, physiological, and metabolic domains. ApoA2‑i function as a central biomarker platform linking four major clinical areas in pancreatic disease. *Early detection*: decreased apoA2‑ATQ/AT improves identification of stage 0–I pancreatic ductal adenocarcinoma (PDAC) and complements CA19‑9 in high‑risk surveillance. *IPMN risk stratification*: reduced ATQ/AT reflects tumor‑related proteolytic alterations and outperforms CA19‑9 for identifying intraductal papillary mucinous neoplasms (IPMNs) with high‑risk stigmata or worrisome features. *Exocrine physiology*: apoA2‑AT/AT serves as a functional marker of pancreatic exocrine insufficiency (PEI), predicting postoperative steatotic liver disease (SLD) when decreased and postoperative pancreatic fistula (POPF) risk when elevated. *NOD → type 3c diabetes stratification*: glycemically defined new‑onset diabetes (GNOD) precedes PDAC by approximately eight months and carries a markedly elevated 3‑year cancer incidence; apoA2‑i, together with fecal elastase‑1 and pancreatic morphology, supports stratification of individuals with emerging type 3c diabetes mellitus (T3cDM). This integrated framework highlights the versatility of apoA2‑i across tumor biology, exocrine function, and metabolic risk assessment.

## 4 Early detection of pancreatic ductal adenocarcinoma

Carbohydrate antigen 19‑9 (CA19‑9) is the most widely used serum biomarker for PDAC; however, its performance is limited by low sensitivity for stage I disease, false‑negative results in Lewis antigen–negative individuals, and frequent elevation in benign conditions such as cholangitis or obstructive jaundice (10). Consequently, CA19‑9 is inadequate as a standalone tool for early detection or for distinguishing PDAC from inflammatory pancreatic diseases. Given these limitations, current early detection strategies focus on individuals at increased risk, including those with hereditary predisposition syndromes, a strong family history, or pancreatic cystic lesions such as IPMNs. Even in these high‑risk groups, imaging‑based surveillance using MRCP or EUS has variable sensitivity, and subtle parenchymal or ductal changes may be difficult to interpret. These challenges underscore the need for minimally invasive biomarkers that can complement imaging and detect biological alterations associated with early‑stage disease.

Recent advances in cancer interception have emphasized the importance of identifying neoplastic disease at presymptomatic stages. Foundational work by Esserman et al. proposed shifting from late‑stage diagnosis toward proactive detection strategies (11), and multianalyte blood tests have demonstrated the feasibility of detecting surgically resectable cancers through circulating biomarkers (12). Within this framework, apolipoprotein A2 isoforms (apoA2‑i) have emerged as promising candidates for early PDAC detection. A large multicenter clinical performance study demonstrated that apoA2‑ATQ/AT achieved an AUC of 0.879, and showed numerically higher performance than CA19-9 for stage I detection, although formal statistical superiority was not consistently demonstrated (13). Subsequent studies confirmed its complementary value alongside CA19‑9 (14) and validated its reproducibility in real‑world and blinded reference‑set analyses (15).

In addition to these ELISA-based multicenter studies, the foundational multi-institutional validation study by Honda et al. provides important methodological context supporting the robustness of apolipoprotein-based biomarkers (24). This study analyzed 1, 314 plasma and serum samples across four cohorts collected from eight medical institutions in Japan and Germany, including pancreatic ductal adenocarcinoma, chronic pancreatitis, other gastrointestinal cancers, and healthy controls. The study design incorporated both retrospective cohorts (Cohorts 1–3) and a large prospective cohort (Cohort 4; n = 833), with blood samples collected under a unified standard operating procedure distributed to all participating institutions. All samples were blinded and randomized prior to mass spectrometry analysis, and measurements were independently reproduced at two laboratories to ensure analytical consistency. Cut-off values for biomarker discrimination were determined using ROC analyses within discovery cohorts and applied to validation cohorts. This multi-institutional framework—spanning prospective specimen collection, blinded evaluation, and independent laboratory replication—provides a methodological foundation for subsequent ELISA-based apoA2 isoform studies and supports the reliability of apoA2-related biomarker signatures in diverse clinical settings.

Independent evidence from a Japanese cohort further supports the utility of apoA2‑isoforms in the earliest detectable phases of pancreatic cancer. Hanada et al. reported that the apoA2-ATQ/AT showed higher sensitivity than CA19-9 for identifying stage 0 and IA pancreatic cancer, although the study did not report formal statistical comparison of AUC values (25). Although this study did not evaluate IPMN dysplasia grades, it provides external validation that apoA2‑i may detect biological alterations associated with very early‑stage pancreatic cancer, and complements CA19‑9 in early cancer detection. Together, these findings highlight the potential of apoA2‑i as a blood‑based biomarker platform capable of supporting risk assessment in high‑risk individuals, improving early detection performance, and aligning with the broader goals of pancreatic cancer interception.

## 5 Comparison with other emerging blood‑ and urine‑based biomarkers

Beyond apoA2‑isoforms, multiple non‑invasive biomarkers have been investigated for early detection of PDAC (**Table 1**). CA19‑9 remains the most widely used serum biomarker for pancreatic cancer, with diagnostic performance consistently superior to other conventional markers. Across multiple clinical studies, CA19‑9 demonstrates an AUC of 0.70–0.80, with sensitivity of 60–70% and specificity of 80–90% for PDAC detection (26). However, its utility is limited by false‑negative results in Lewis antigen‑negative individuals and elevations in benign inflammatory conditions, reducing its effectiveness as a standalone early detection tool. Carcinoembryonic antigen (CEA) has historically been used as an adjunctive serum biomarker for pancreatic cancer. A systematic review and meta‑analysis reported a pooled sensitivity of 45% and specificity of 89% for CEA in PDAC, indicating limited diagnostic utility when used alone (27). DUPAN‑2, a sialylated glycan biomarker frequently used in Japan, has shown improved diagnostic performance when reference ranges are individualized according to FUT2/FUT3 genotypes. Although the recent high‑impact study evaluating genotype‑adjusted DUPAN‑2 cutoffs was conducted in a US cohort, it demonstrated a sensitivity of 60.4% and specificity of ~99% for stage I/II PDAC, with further enhancement when combined with CA19‑9 (sensitivity 78.4%, specificity 97.7%) (28). Blood‑based assays such as circulating tumor DNA (ctDNA), circulating tumor cells (CTCs), exosomal cargo, and multi‑analyte protein panels have demonstrated varying degrees of diagnostic performance, although sensitivity in stage 0–I disease remains inconsistent across studies. For ctDNA, KRAS mutations are detected in more than 90% of metastatic PDAC cases but only ~48% of localized tumors, reflecting limited shedding of tumor DNA in early disease (29). Fragment‑size–based enrichment strategies can improve ctDNA detection, but their clinical utility in early PDAC remains under investigation (30). Recent comprehensive reviews have highlighted the growing clinical relevance of liquid biopsy components including CTCs, cfDNA/ctDNA, miRNAs, extracellular vesicles (EVs), and tumor‑educated platelets (TEPs), particularly for prognosis and treatment monitoring rather than early detection (31).

**Table 1:** Representative blood‑ and urine‑based biomarkers evaluated for early detection of PDAC.

Biomarker	Specimen	Representative study	AUC	Sensitivity	Specificity	Notes
ApoA2 isoforms	Blood	Multicenter studies (Japan)	0.80–0.90	70–80%	80–90%	Promising performance in selected cohorts, including IPMN and early-stage PDAC, although sensitivity varies by stage and remains investigational. Independent of Lewis antigen status.
CA19-9	Blood	Standard clinical studies	0.70–0.80	60–70%	80–90%	Limited by false negatives in Lewis-negative individuals; elevated in inflammation.
CEA	Blood	Meng et al., 2017 (meta‑analysis)	~0.60	40–50%	70–80%	Low sensitivity for pancreatic cancer; mainly adjunctive.
DUPAN-2	Blood	Ando et al., JCO 2024(US cohort)	0.65–0.75	50–60%	70–80%	Often used as a complementary marker to CA19-9.
THBS2 + CA19-9	Blood	Sci Transl Med 2017	0.87	82%	92%	Improved performance in stage I pancreatic cancer.
sAXL + CA19-9	Blood	eBioMedicine 2022	0.83–0.90	75–80%	85–90%	Improves PDAC–CP discrimination; validated in two independent cohorts.
Glypican‑1 positive exosomes	Blood	Nature 2015	≥0.90	80–90%	80–90%	High reported accuracy; reproducibility remains debated.
cfDNA/ctDNA(KRAS mutations)	Blood	Multicentre studies	0.75–0.85	60–70%	80–90%	Lower detection rates in early-stage disease.
Urinary LYVE1/REG1B/TFF1	Urine	Debernardi et al., IJC 2023	0.90	83%	87%	Non-invasive; strong performance even in IPMN.
LYVE1/REG1B + CA19‑9	Urine + Blood	Tajbakhsh et al., J Mol Diagn 2025	0.89	83%	87%	Analytical and clinical validation in a large cohort of high‑risk vs stage I–II cancer patients

In addition to blood‑based biomarkers, urine‑based protein signatures have emerged as promising non‑invasive tools for early PDAC detection. A large nested case‑control study by Debernardi et al. evaluated the urinary panel consisting of LYVE1, REG1B, and TFF1 in prediagnostic samples collected up to five years before PDAC diagnosis (32). In this prospective cohort analysis, the PancRISK algorithm—integrating the three urinary proteins with creatinine and age—achieved an AUC of 0.79 within one year before diagnosis and maintained discriminative ability up to two years. Importantly, when combined with plasma CA19‑9, the model reached an AUC of 0.89 within one year and 0.77 within two years, demonstrating substantial improvement over either biomarker alone.

A CLIA‑compatible ELISA using only LYVE1 and REG1B in combination with CA19‑9 has recently been developed, demonstrating similar performance in a 565‑patient cohort. This assay distinguished early‑stage resectable pancreatic cancer from pooled asymptomatic and symptomatic non‑cancer patients (including 136 pancreatitis and 73 benign cysts), achieving an AUC of 0.89 with 83% sensitivity and 87% specificity (33).

Several blood‑based multi‑analyte panels have also shown enhanced performance compared with CA19‑9 alone. The combination of thrombospondin‑2 (THBS2) and CA19‑9 demonstrated an AUC of 0.87 for stage I PDAC (34), while soluble AXL (sAXL) combined with CA19‑9 achieved AUC values of 0.83–0.90 in early‑stage disease (35). Exosomal glypican‑1 has been reported to reach AUC values ≥0.90 in early PDAC detection, although reproducibility remains debated (36).

To contextualize apoA2‑isoforms within this broader biomarker landscape, **Table 1** summarizes representative blood‑ and urine‑based biomarkers evaluated in early detection studies. As shown, apoA2‑i exhibit comparable AUC values to several emerging liquid biopsy platforms, particularly in early‑stage disease, while providing the unique advantage of reflecting pancreatic exocrine physiology rather than tumor‑derived genomic alterations. These distinctions suggest that apoA2‑i may integrate effectively with tumor‑centric liquid biopsy assays in multimodal diagnostic algorithms.

## 6 Apolipoprotein A2 isoforms as blood‑based biomarkers in pancreatic oncology

### 6.1 Diagnostic utility for PDAC and IPMNs

ApoA2 isoforms provide diagnostic value across both pancreatic ductal adenocarcinoma (PDAC) and intraductal papillary mucinous neoplasms (IPMNs). Their utility extends beyond early detection and offers clinically meaningful stratification of cystic neoplasms, particularly when integrated with imaging-based surveillance.

Building on these diagnostic observations, the apoA2‑ATQ/AT isoform serves as a key blood‑based indicator of malignant potential in IPMNs. Multiple independent studies have demonstrated that ATQ/AT provides strong discriminatory ability for identifying IPMNs with high‑risk stigmata or high‑grade dysplasia, with numerically higher performance than CA19‑9 and complementing imaging‑based surveillance (16, 25).

Hasegawa et al. reported that apoA2‑ATQ/AT demonstrated significantly better discriminatory ability than CA19‑9 for IPMN risk stratification. This recent single‑center retrospective study evaluated apoA2‑i in 212 patients with branch‑duct IPMN, categorized by EUS findings into high‑risk stigmata (HRS), worrisome features (WF), and non‑WF groups, together with 295 age‑ and sex‑matched healthy volunteers. Plasma apoA2‑i was quantified using a validated ELISA, and diagnostic performance was assessed using ROC analyses with formal statistical comparison of AUCs (DeLong test). ApoA2‑i demonstrated significantly better discrimination of HRS and WF lesions compared with CA19‑9 (AUC 0.676 vs 0.554; p=0.029) whereas CA19‑9 showed no significant differences across risk groups (16). All biomarker measurements and radiologic assessments were performed in a blinded manner. Although these findings indicate superior performance of apoA2‑i in this cohort, further multicenter prospective validation will be required before clinical integration.

Of note, the latest international consensus guidelines for IPMN, the 2024 Kyoto Guidelines, recommend CA19‑9 as the only blood‑based biomarker incorporated into risk stratification algorithms (37). Thus, although CA19‑9 is included in the guideline algorithm, its diagnostic performance is limited, and it is not recommended as a definitive biomarker for IPMN risk stratification.

Given that apoA2‑ATQ/AT demonstrates s promising discriminatory ability compared with CA19‑9, these findings suggest that apoA2‑isoform testing may warrant consideration in future research evaluating potential biomarker integration into guideline frameworks, although formal incorporation would require multicenter, prospective validation. Because ATQ/AT is consistently decreased across both hyperprocessing and hypoprocessing patterns, its reduction reflects proteolytic and microenvironmental alterations associated with neoplastic progression. These characteristics position apoA2‑ATQ/AT as a biologically grounded and clinically clinically supported but still investigational biomarker for IPMN risk stratification, with potential to contribute to earlier identification of lesions that may warrant surgical evaluation.

### 6.2 ApoA2‑i as functional biomarkers reflecting pancreatic exocrine physiology

A unique strength of apoA2‑i is their ability to reflect pancreatic exocrine function, offering insights into postoperative physiology that traditional tumor markers cannot provide. The light isoform, apoA2‑AT/AT, decreases in the setting of pancreatic exocrine insufficiency (PEI), consistent with reduced carboxypeptidase activity and impaired digestive enzyme secretion (23). This biological relationship enables apoA2-i to serve as potential functional biomarkers in perioperative management.

Postoperative reductions in apoA2‑AT/AT have been shown to identify patients at high risk for developing new-onset steatotic liver disease (SLD) following pancreatectomy (17). ApoA2-AT/AT levels <9.58 μg/mL, the median value in this cohort, were significantly associated with SLD (*p* < 0.001) and were an independent risk factor for SLD in both univariable analysis (odds ratio [OR], 20.1; 95% confidence interval [CI], 6.59–74.5; *p* < 0.001) and multivariable logistic regression analysis (OR, 13.6; 95% CI, 2.68–134.2; *p* = 0.001). This study was a single‑center retrospective analysis of 79 patients undergoing pancreatectomy. Postoperative apoA2-AT/AT levels were evaluated as functional biomarkers of pancreatic exocrine physiology. Radiologic assessment of SLD was performed independently and blinded to biomarker data. These findings indicate that apoA2‑i may noninvasively monitor postoperative pancreatic exocrine function and could guide enzyme replacement therapy to prevent malnutrition‑associated hepatic injury, although further prospective multicenter study is required.

Conversely, elevated preoperative apoA2‑AT/AT levels reflect preserved acinar activity and increased secretion pressure at the pancreatic stump. Ono et al. reported that apoA2‑AT/AT ≥58.17 µg/mL was independently associated with postoperative pancreatic fistula (POPF) following distal pancreatectomy, with an AUC of 0.802 for prediction (16). The distal pancreatectomy cohort reported was a single-center retrospective study of 51 patients. Preoperative apoA2-AT/AT was measured using ELISA, and the optimal cut-off (58.17 μg/mL) was determined by ROC analysis. POPF grading followed ISGPS criteria, and outcome assessors were blinded to biomarker measurements. This suggests that apoA2-i may contribute to preoperative risk stratification and inform perioperative decision-making, including drain management and postoperative monitoring. As a single-center study, these findings should be interpreted as exploratory and require multicenter validation. Together, these findings demonstrate that apoA2-i function not only as diagnostic biomarkers for pancreatic neoplasia but also as investigational physiological markers of exocrine function, with potential to support personalized perioperative management (**Figure 3**).

### 6.3 Stratification of new‑onset diabetes for type 3c diabetes mellitus based on exocrine function

New‑onset diabetes (NOD) has long been recognized as a potential early manifestation of pancreatic ductal adenocarcinoma (PDAC). A landmark population‑based study demonstrated that individuals who develop diabetes after age 50 have a significantly increased probability of being diagnosed with PDAC within three years, establishing NOD as a clinically meaningful risk group for early detection efforts (38). More recently, a large prospective cohort study using real‑time electronic health record surveillance showed that glycemically defined NOD (GNOD) carries a 3‑year PDAC incidence of 0.62–0.84%, representing a more than ten‑fold increase over the general population. This finding indicates that GNOD precedes clinical cancer diagnosis by a median of 8 months, underscoring its value as a metabolic harbinger of PDAC (39).

Concurrently, advances in the understanding of type 3c diabetes mellitus (T3cDM) have clarified that exocrine pancreatic insufficiency (PEI) is central to its pathophysiology. In a comprehensive review, Hart and colleagues emphasized that endocrine dysfunction progresses in parallel with exocrine failure in both chronic pancreatitis and PDAC, reflecting the shared anatomic and inflammatory milieu of the diseased pancreas (40). Consistent with this, the diagnostic criteria proposed by Ewald and Bretzel designate PEI as a major and required criterion for T3cDM, alongside structural pancreatic abnormalities and the absence of autoimmune markers (41).

Integrating these findings, a biologically grounded concept emerges: within the heterogeneous population of NOD, individuals who exhibit evidence of exocrine dysfunction are more likely to have T3cDM, including PDAC‑associated T3cDM. This provides a potential biologically grounded framework for risk stratification.

Potential markers for identifying exocrine‑driven NOD include:

- Exocrine enzyme activity, such as fecal elastase‑1, and apoA2-i isoforms as investigational exocrine-based markers.
- Pancreatic morphology, such as atrophy, fatty infiltration, and ductal irregularity.
- Metabolic phenotype, including weight loss, malabsorption, and unstable glycemia.
- Microbiome signatures associated with PEI‑related dysbiosis.

In this review, **Figure 3** incorporates this concept by positioning “NOD T3cDM stratification” within the broader clinical applications of apoA2‑i biology, specifically under the domain of exocrine physiology. This placement reflects the mechanistic link between exocrine dysfunction and PDAC‑related metabolic derangements, and highlights the emerging role of exocrine‑based stratification in early detection strategies, although these applications remain exploratory and require prospective validation.

As research continues to refine the metabolic and molecular signatures of PDAC‑associated diabetes, exocrine‑focused stratification of NOD represents a promising and biologically coherent approach to identifying individuals at highest risk, although these applications remain exploratory and require prospective validation.

## 7 Clinical implications and future perspectives

ApoA2‑i have now demonstrated clinical relevance across multiple domains, including early cancer detection, IPMN risk stratification, assessment of exocrine physiology, and the emerging field of NOD‑based stratification for T3cDM. These expanding applications provide several important directions for future research.

ApoA2‑i represent a unique class of blood‑based biomarkers that bridge two traditionally separate domains in pancreatic disease management: early cancer detection and assessment of pancreatic exocrine function. This dual capability distinguishes apoA2‑i from conventional tumor markers such as CA19‑9, which lack sensitivity for early‑stage disease and provide no insight into postoperative physiology. By integrating evidence across early detection, precursor lesion stratification, and perioperative risk assessment, this review highlights apoA2‑i as a biologically grounded and clinically promising but still investigational biomarker platform.

From a diagnostic perspective, apoA2‑i have demonstrated c numerically higher performance over CA19-9 for detecting early-stage PDAC, although statistically significant differences have not been consistently observed across all cohorts, with multicenter studies showing AUC values approaching 0.88 for stage I disease (13). Their performance has been validated across independent cohorts, real‑world clinical settings, and blinded reference sets, underscoring their robustness and reproducibility (14, 15). Notably, apoA2‑i also stratify malignant potential in IPMNs, providing a noninvasive complement to imaging‑based surveillance and addressing a major unmet need in the management of pancreatic cystic lesions (16). These findings align with the broader goals of cancer interception, in which blood‑based biomarkers may support risk assessment in high-risk individuals and enable detection at surgically curable stages.

Equally significant is the emerging role of apoA2‑i as functional biomarkers of pancreatic exocrine physiology. The light isoform, apoA2‑AT/AT, decreases in the setting of PEI, reflecting reduced carboxypeptidase activity and impaired digestive enzyme secretion. This biological relationship provides a mechanistic explanation for recent clinical observations (42). Our group demonstrated that markedly decreased postoperative apoA2‑AT/AT levels identify patients at high risk for new‑onset SLD following pancreatectomy, a complication driven by malnutrition and PEI. Conversely, elevated preoperative apoA2‑AT/AT levels predict POPF after distal pancreatectomy, likely reflecting preserved acinar activity and increased secretion pressure at the pancreatic stump. Together, these findings indicate that apoA2‑i may reflect dynamic physiological changes that are not reflected by traditional tumor markers or imaging studies. Moreover, because postoperative steatotic liver disease and maldigestion arise from varying degrees of pancreatic exocrine insufficiency, apoA2‑i may help guide the appropriate dosing of pancreatic enzyme replacement therapy by providing a blood‑based indicator of residual exocrine function, although prospective validation is needed.

The convergence of these diagnostic and functional properties positions apoA2‑i as a comprehensive biomarker platform capable of informing clinical decision‑making across the continuum of pancreatic disease. For early detection, apoA2‑i may contribute to identifying individuals who could benefit from targeted imaging. For IPMN surveillance, apoA2‑i may help distinguish lesions requiring surgical intervention from those suitable for observation. In the perioperative setting, apoA2‑i may guide enzyme replacement therapy, identify patients at risk for SLD, and support individualized strategies to mitigate POPF risk, although these applications remain exploratory.

Future research should focus on integrating apoA2‑i into multimodal diagnostic algorithms, that combine blood‑based biomarkers with imaging, genomics, and clinical risk factors. Prospective validation in diverse populations, including high‑risk cohorts and postoperative patients, will be essential for establishing standardized cutoff values and clinical workflows. Additionally, mechanistic studies exploring the proteolytic processing of apoA2‑i may further elucidate their relationship to pancreatic physiology and tumor biology.

Although apoA2 isoforms show promise across diagnostic and physiological domains, several limitations should be acknowledged. Current evidence for metabolic applications—particularly the stratification of new‑onset diabetes for identifying type 3c diabetes mellitus—remains preliminary and requires prospective validation. Standardized cutoff values, assay harmonization, and integration into clinical workflows have not yet been established. In addition, mechanistic studies are needed to clarify how specific exocrine phenotypes influence apoA2 isoform processing. Addressing these gaps will be essential for translating apoA2‑based approaches into routine clinical practice.

In summary, apoA2‑i offer a rare combination of diagnostic precision and functional insight, enabling an integrated approach to early detection, risk stratification, and perioperative management in pancreatic oncology. Their integration into clinical practice has the potential to shift the paradigm toward biology‑driven, personalized care, pending further multicentre validation.

## 8 Conclusion

Apolipoprotein A2 isoforms (apoA2‑i) provide an integrated biomarker perspective that connects the biological foundations of pancreatic cancer with practical clinical decision‑making. By reflecting both neoplastic transformation and pancreatic exocrine dysfunction, apoA2‑i may complement the limitations of conventional tumor markers and offer investigational value across early detection, precursor lesion stratification, and perioperative risk assessment. Their diagnostic precision for early‑stage PDAC, ability to identify high‑risk IPMNs, and capacity to reflect postoperative exocrine physiology, including risks of steatotic liver disease and pancreatic fistula, highlight their potential versatility as blood‑based indicators of disease biology. As evidence continues to expand, integrating apoA2‑i into multimodal diagnostic and perioperative algorithms could contribute to a more biology‑driven, personalized approach to pancreatic cancer interception and management, although prospective multicener validation, assay harmonization, and standardized clinical workflows will be essential before routine implementation.
